# Wentilactone A Reverses the NF-κB/ECM1 Signaling-Induced Cisplatin Resistance through Inhibition of IKK/IκB in Ovarian Cancer Cells

**DOI:** 10.3390/nu14183790

**Published:** 2022-09-14

**Authors:** Cuiting Lv, Chunxia Ren, Yinjue Yu, Huijing Yin, Caiguo Huang, Gong Yang, Yang Hong

**Affiliations:** 1 Central Laboratory, The Fifth People’s Hospital of Shanghai, Fudan University, Shanghai 200240, China; 2 Center for Reproductive Medicine, Shuguang Hospital Affiliated to Shanghai University of Traditional Chinese Medicine, Shanghai 201203, China; 3 Department of Radiotherapy, Ren Ji Hospital, School of Medicine, Shanghai Jiao Tong University, Shanghai 200127, China; 4 Cancer Institute, Fudan University Shanghai Cancer Center, Shanghai 200032, China; 5 Department of Biochemistry and Molecular Biology, College of Basic Medical, Navy Medical University, Shanghai 200433, China; 6 Department of Oncology, Shanghai Medical School, Fudan University, Shanghai 200032, China; 7 Department of Orthopedics, The Fifth People’s Hospital of Shanghai, Fudan University, Shanghai 200240, China

**Keywords:** NF-κB/ECM1, tumor microenvironment, cisplatin resistance, wentilactone A, ovarian cancer

## Abstract

Wentilactone A (WA) is a tetranorditerpenoid isolated from marine algae. We previously found that WA inhibited cancer cell proliferation with little toxicity. In this study, we show that high expression of extracellular matrix protein-1 (ECM1) promotes cancer cell cisplatin resistance, and the secreted ECM1 activates normal fibroblasts (NFs) to transform cells with characteristics of cancer-associated fibroblasts (CAFs). Transcription of the ECM1 gene is regulated largely by NF-κB through EP881C/T-EP266C binding sites. WA supresses the phosphorylation of NF-κB through inhibition of the upstream IKK/IκB phoshorylation to block the expression of ECM1, which reverses the cisplatin-induced activation of NF-κB/ECM1. On the contrary, cisplatin facilitates phosphorylation of NF-κB to enhance the expression of ECM1. These results highlight ECM1 as a potential target for treatment of cisplatin-resistant cancers associated with the ECM1 activated signaling. In addition, WA reverses cisplatin resistance by targeting both tumor cells and the tumor microenvironment through IKK/IκB/NF-κB signaling to reduce the expression of the ECM1 protein.

## 1. Introduction

Ovarian cancer is a high-risk malignant gynecological tumor with high chemoresistance and mortality [1,2]. Although the platinum-based two-drug combination regimen is the first-line therapy after surgery, 70% of patients undergo drug resistance, metastasize and eventually relapse [3]. Malignant chemo-resistance is a phenomenon of cells or tissues adapting to damaging chemicals. Most first-line chemotherapeutic drugs induce a bypass effect, thereby weakening the efficacy of the drug, ultimately leading to treatment failure and tumor recurrence [4]. Activation of the NF-κB pathway is an important factor in cisplatin (CDDP) resistance [5]. Cisplatin may activate the MEK/ERK signaling pathway, phosphorylate and ubiquitinate IκB to increase the transcription activity of NF-κB [6]. There is no effective adjuvant that can efficiently reverse the resistance of ovarian cancer to cisplatin, and the targeted therapy cannot replace or cooperate with cisplatin to improve the survival rate.

The tumor microenvironment also participates in the chemo-therapeutic response [7]. Soluble factors secreted by cancer or stromal cells can induce microenvironment-mediated drug resistance. Among these factors, extracellular matrix proteins are critical in tumor microenvironment remodeling to facilitate tumor progression and metastasis [8]. Rational drugs targeting both tumor and microenvironmental cells might be more sufficient to overcome chemo-resistance [9].

The human *Extracellular matrix 1* (ECM1) is located on chromosome lq21 and contains 11 exons encoding a secretory glycoprotein [10]. The ECM1 protein has four splicing variants: ECM1a, ECM1b, ECM1c, and ECM1d [10,11]. Most of the early functional studies on ECM1 are focused on bone and cartilage development, skin formation, and angiogenesis, other than malignancies [12,13,14]. Recent findings suggest that ECM1 is associated with cancer cell migration, invasion, adhesion, and angiogenesis [15,16,17]. Highly expressed ECM1 in a variety of malignant epithelial cells is correlated with metastasis and poor prognosis [14,18]. Nevertheless, the putative regulators of ECM1 at the transcriptional level have not been identified. As the component of the extracellular matrix (ECM), the detailed mechanism associated with the ECM1-mediated tumor microenvironment remodeling is also unclear.

It is well known that marine natural products may be developed as anti-cancer agents. We previously reported that asperolides A-C and five related derivatives, isolated from the culture extract of an endophytic fungus (*Aspergillus wentii EN-48*) from an unidentified marine brown algal *Sargassum*, displayed cytotoxic activity against various human cancer cells [19,20,21]. The endophytic fungus *Aspergillus wentii EN-48* isolated from the fresh tissue of *Sargassum* that are commonly consumed as food and traditional medicine in Asian countries [22]. *Sargassum* also presents antipyretic, analgesic, antimicrobial, antiedema, antioxidant, antitumor, anti-inflammatory, and hepatoprotective activities [23,24]. Moreover, *Sargassum* is a source for high-value secondary metabolites with potential benefits for human health, such as fucoidans and alginates that have received great attention in the health industry. The nutritional composition also makes this alga suitable food [25,26]. Herein, we show that a secondary metabolite WA from *Sargassum* reverses the cisplatin resistance of ovarian cancer cells through inhibition of the NF-κB-activated signaling pathway to induce the expression of ECM1. ECM1 is associated not only with cisplatin resistance of cancer cells, but also with the malignant transformation of NFs into CAFs. Therefore, WA reverses cisplatin resistance by targeting both tumor and microenvironmental cells.

## 2. Materials and Methods

### 2.1. Chemical Compound and Preparation

WA was isolated from the culture of the *Aspergillus wentii EN-48* fungal strain and was supplied by the Institute of Oceanology of the Chinese Academy of Sciences [19]. It was dissolved in DMSO and stored at −20 °C, then diluted to different concentrations with cell culture medium before using. The final DMSO concentration is less than 0.1%.

### 2.2. Cell Culture

Human ovarian epithelial cancer cell lines SKOV3, SKOV3ip1, OVCA429, OVCA433, Hey, Hey-A8 (A8), A2780, cisplatin resistant A2780cis cell line, normal human ovarian epithelial HOSE cell line, normal fibroblasts (NFs), and cancer-associated fibroblasts (CAFs) were obtained from the laboratory of Fudan University Shanghai Cancer Center [27,28]. These cell lines were identified according to the STR data, and were routinely tested and found to be free of mycoplasma. All cells were cultured with RPMI1640 media added with 10% fetal bovine serum, 100 U/mL penicillin, and 100 μg/mL streptomycin at 37 °C in a humidified 5% CO_2_ atmosphere.

### 2.3. Establishment of Cell Lines by Gene Silencing or Over-Expression

To knock down ECM1 expression, shRNAs (ECM1-sh-FW, 5′-ccggAGAGCCATCCAGAACCTGAGTctcgagACTCAGGTTCTGGATGGCTCTtttttg-3′; ECM1-sh-RV, 5′-aattcaaaaaaAGAGCCATCCAGAACCTGAGTctcgagACTCAGGTTCTGGATGGCTCT-3′) targeting the human ECM1 mRNA sequence were inserted into the lentiviral vector plko.1/puromycin [29]. cDNAs for ECM1 isoforms a, b, and c were amplified from A8 cells by RT-PCR using the forward (ECM1-FW, 5′-ataatTCTAGAGCTAGCGAATTCatggggaccacagccagagcag-3’) and reverse primer(ECM1-RV, 5′-ataatGCGGCCGCGGATCCtcaAGCGTAGTCTGGGACGTCGTATGGGTAttcttccttgggctcagag-3’) with hemagglutinin (HA) tag. Each of the ECM1 subtype cDNAs was cloned into pCDH/neomycin lentiviral vector obtained from Addgene System Biosciences.

The plasmids were co-transfected with packaging vectors psPAX2 and pMD2.G at a ratio of 4:3:1.2 into HEK293T cells and harvested at 24 h, 48 h and 72 h. The harvested supernatant was used to infect target cells with polybrene (10 μg/mL). The target cells were infected for two days, and then selected with puromycin or neomycin for 14 days to establish new stable cell lines.

### 2.4. Cell Counting Kit-8 (CCK-8) Assay

The CCK-8 assay was used to examine the in vitro cytotoxicity of WA and cisplatin resistance. Four thousand cells were seeded in each well of 96-well plates and cultured for 24 h, and then treated with diluents or different concentrations of drugs for 48 h. After that, 10 μL WST^®^-8 (Dojindo, Japan) was added in each well, followed by incubation for 1 h at 37 °C. The absorbance value was detected with a microplate reader at a wavelength of 450 nm. The inhibition ratio was calculated using the following formula: Inhibition ratio = (OD control − OD treatment)/(OD control − OD blank). The IC_50_ was calculated using GraphPad Prism.

### 2.5. Western Blotting Analysis

Cell lysates were collected using RIPA buffer (Beyotime, Nantong, China) added with a protease inhibitor, and then the different proteins were detected by a standard WB procedure [30]. The primary antibody to ECM1 (ab126629) was from Abcam (Cambridge, England, UK). The antibodies to p-IKKα/β (Ser176/180, #2697), p-IκBα (Ser32, #2859), NF-κB p65 (#6956), p-NF-κB p65 (Ser 536, #3033), snail (#3879), FAP (#66562) and α-SMA (#19245) were from Cell Signaling Technology (CST, Danvers, MA, USA). The secondary antibodies against mouse (115-035-003) or rabbit IgG (111-035-003) were purchased from Jackson Immuno Research (West Grove, PA, US). The immunoblots were visualized by a chemoluminescence reagent (Millipore, Burlington, MA, USA) and detected on Fluorchem E from Protein Simple (San Jose, CA, USA).

### 2.6. Mouse Xenograft Model

The animal assay was designed with reference to published articles [20,31] and approved by the Institutional Animal Care and Use Committee of East China Normal University (Animal ethics was approved in January 2019 with the Approval Number AR2020136). 3 × 10^6^ A2780cis or ECM1 silenced A2780cis cells were subcutaneously (s.c.) injected into the right armpit of five-week old BALB/c female athymic mice. Administration began after the average tumor sizes reached 50 mm^3^. The mice were randomized into four groups (four mice per group): vehicle control (1% DMSO), 3 mg/kg cisplatin, 5 mg/kg WA, and cisplatin + WA. Vehicle-treated mice were used as negative control, and cisplatin-treated mice were used as positive control to assess the effect of WA combination. Intraperitoneal injection of vehicle or drugs was performed every five days; body weight and tumor size were recorded at the same time. The tumor volumes were calculated by using the formula: length × width^2^/2. Mice were sacrificed on the 20th day of administration; tumors were collected, weighed, and photographed. The equation: tumor suppression (%) = (1 − T/C) × 100 was used to calculate tumor inhibitory rates, where T is the average tumor weight of the treated group and C is that of the control group.

### 2.7. ECM1 Promoter Cloning

The wild-type ECM1 promoter (−1149 to −1) containing the putative binding site for p65 was amplified using normal human DNA as a template and cloned into the pGL3-Basic (Promega, Madison, WI, USA) using the primers in Table 1. Two binding motifs, GGGagacCCC at −890 to −881 (named EP881C) and GGGagatCCC at −275 to −266 (named EP266), were predicted according to the consensus sequence of GGGRNNYCCC (R-purine, N-any nucleotide, and Y-pyrimidine), as reported elsewhere [32]. In addition, a C/T SNP was found (GGGagacCCT) at −890 to −881 (named EP881T). Therefore, site-directed mutagenesis of the predicted consensus sequences was performed using a Quik Change kit (#200523, Stratagene, San Diego, CA, USA) and primers 3–8 (Table 1) to obtain the single-motif mutation (TTT-EP881C, TTT-EP881T, and TTT-EP266) or double-motif mutation (TTT-EP881C/TTT-EP266 and TTT-EP881T/TTT-EP266). HIV WT or mutant promoters that were previously reported to bind to NF-κB were used as positive and negative controls [33].

### 2.8. Luciferase Assay

To perform luciferase assays driven by the ECM1 promoter, ovarian cancer cells (A8) were plated in 96-well plates and transiently transfected with 200 ng of pGL3 or NF-κB plasmid each well using Lipofectamine 2000. The cells were also co-transfected with 10 ng of a construct encoding Renilla luciferase to normalize for transfection efficiency. After 48 h of transfection, luciferase activity was measured according to the protocol from the Dual-Luciferase Assay kit (Promega, Madison, WI, USA). Firefly luciferase activity was standardized to Renilla luciferase activity. Triplicate samples were assayed three times.

### 2.9. Immunofluorescence Staining

Immunofluorescence staining was completed following a published protocol [34]. A2780cis cells were seeded on the slides and treated with 15 μM cisplatin, 0.5 μM WA alone, or combination for 24 h and then fixed in methanol for 5 min, permeabilized for 5 min with 0.3% Triton X-100 in PBS, blocked for 1 h with 5% bovine serum albumin, then incubated with p-p65 (Ser 536) (1:150) antibody overnight at 4 °C. The next day, cells were washed and incubated with secondary antibodies conjugated with Alexa Fluor 594 (Jackson Immuno Research, West Grove, PA, USA) in a dark humid box at ambient temperature for 1 h, then washed again and stained with DAPI. All stained cells were calculated and photographed with a fluorescent microscope camera.

### 2.10. Cell Culture Conditioned Medium (CM) and Detection of Secreted ECM1

Cells cultured at 80% confluence were fed with fresh medium. After 24 h, the supernatant was collected as conditioned medium (CM). The CM was centrifuged using Amicon^®^ Ultra-15 Centrifugal Filters (ufc903096, Millipore, Billerica, MA, USA), and then 5× protein loading dye (Sango Biotech, Shanghai, China) was added to obtain protein lysates for ECM1 detection by western blot.

### 2.11. 3D Spheroid Formation Assay

Rat tail collagen (#354236, Corning, New York, NY, USA) was diluted to 50 µg/mL with 0.02 N acetic acid. 500 μL diluted material was added to each well on a 24-well plate. Then plate was incubated at ambient temperature for 1 h. The remaining solution was aspirate, and the wells were rinsed with PBS to remove acid. The cancer cells and normal fibroblasts (NFs) at the ratio of 1:50 or cancer cells alone were mixed with 1640 medium to get a cell suspension of 250 cells/500 μL. The cell suspension was mixed with a solution of 1640 medium, material and NaOH at the ratio of 5:1:0.0235 on ice, then the mixture was plated in the wells. After 7 days’ incubation, the spheroid number was counted under a microscope.

### 2.12. Immunohistochemistry Staining

Xenograft tumor samples were fixed in 10% formalin and embedded in paraffin wax. 4-mm sections were cut from the paraffin blocks. The sections were then stained for 6 h at 4 °C with anti-ECM1 (1:150) or anti-p65 (1:150). The secondary antibody against mouse/rabbit IgG was from an EnVision™ Detection Kit (GENE, San Francisco, CA, USA). For coloration, the dark brown color by Diaminobenzidine (DAB) represents a strong positive staining.

### 2.13. Statistical Analysis

The data is expressed as the mean ± SD. Comparisons among groups were analyzed using a Student’s *t*-test and ANOVA of GraphPad Prism software. *p* < 0.05 was considered statistically significant (* refers to *p* < 0.05; ** refers to *p* < 0.01; *** refers to *p* < 0.001).

## 3. Results

### 3.1. WA Reverses Cisplatin Resistance in ECM1 Highly-Expressed Ovarian Cancer Cells

**Figure 1:**
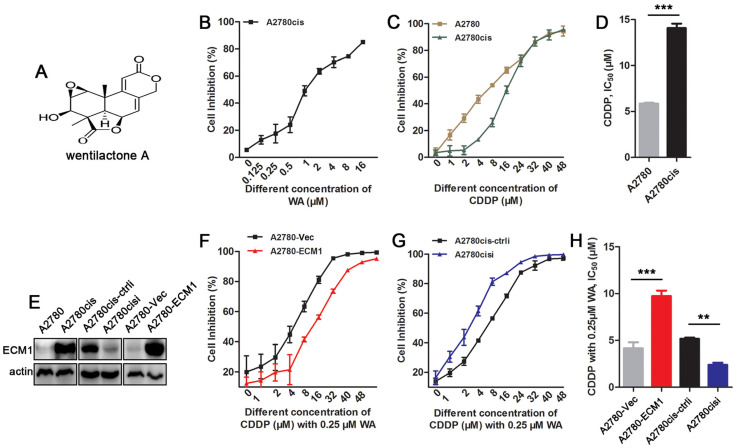
WA inhibits ECM1-mediated cisplatin resistance in cisplatin resistant ovarian cancer cells. The chemical structure of compound (**A**). The inhibition rates of cells treated with WA (**B**) or cisplatin (**C**) analyzed by CCK8 assay after 48 h. (**D**) Cisplatin IC_50_ values of A2780cis and A2780 cells. (**E**) ECM1 expression in A2780, A2780cis and their derivatives were detected by western blot. β-actin is used as a loading control. Inhibition curves (**F**,**G**) and cisplatin IC_50_ values (**H**) of above cell lines treated with cisplatin and WA (0.25 µM). Means ± SD, *n* =  3. ** *p* < 0.01, *** *p* < 0.001. “i” means ECM1 interfering RNA, “ctrli” stands for control of ECM1 interfering RNA, “Vec” stands for empty vector, “CDDP” stands for cisplatin.

The chemical structure of WA is shown in Figure 1A. We first examined the WA and CDDP sensitivity of A2780 and cisplatin resistant ovarian cancer cell line A2780cis using CCK-8 assay. As shown in Figure 1B, treatment of A2780cis cells with 0–16 µM WA for 48 h highly reduced the cell proliferation in a dose-dependent manner. The IC_50_ values of A2780 and A2780cis cells were 5.85 µM and 14.03 µM, respectively, after cells treated with 0–48 µM CDDP (Figure 1C,D). Based on our previous study [27], ECM1 is associated with CDDP resistance. Therefore, we tested the expression of ECM1 in A2780 and A2780cis cell lines, and found that the expression of ECM1 in A2780cis was higher than that in A2780 cells (Figure 1E and Figure S1). We further silenced the expression of ECM1 in A2780cis cells and overexpressed ECM1a in A2780 cells (hereafter labeled as “A2780-ECM1”). The silencing and overexpression of ECM1 were confirmed by western blot analysis (Figure 1E and Figure S1). The CCK-8 assay showed that the IC_50_ of A2780cis cells was reduced to 5.18 µM by the combination of 0.25 µM WA (less than 20% inhibition concentration) and CDDP. When ECM1 was silenced in A2780cis, the IC_50_ value of the cells was further reduced to 2.37 µM, whereas overexpression of ECM1 in A2780 increased the IC_50_ value to 9.73 µM compared with that of control (4.14 µM) (Figure 1F–H).

### 3.2. Resistent Reversal Efficacy of WA + Cisplatin Is Associated with ECM1 Expression

**Figure 2:**
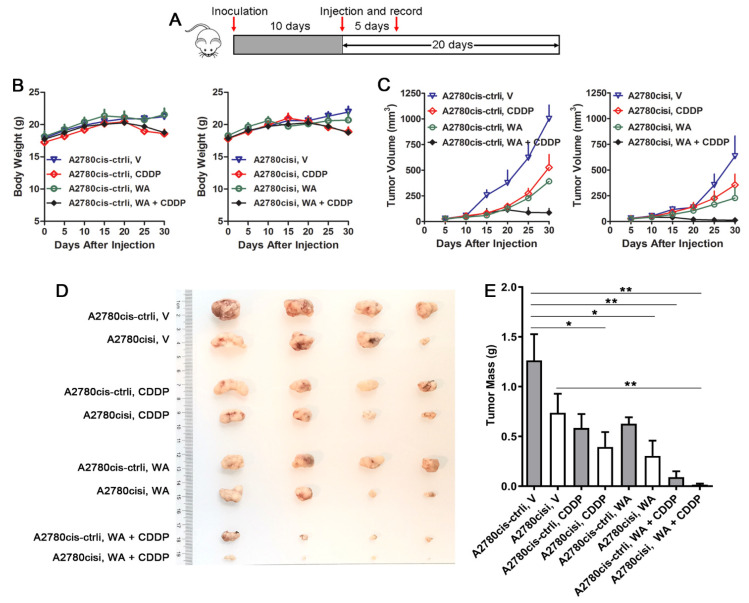
Tumor growth after treatment with CDDP and/or WA. Mice were treated with vehicle (V, 1% DMSO) or drugs according to the panel (**A**). (**B**) Body weight of nude mice. (**C**) Tumor growth induced by A2780cis cells expressing vector (A2780cis-ctrli) or ECM1 shRNA (A2780cisi), followed by administration of vehicle, 3 mg/kg cisplatin, 5 mg/kg WA, or cisplatin + WA. (**D**) Tumor images from various treatment groups. (**E**) Average tumor mass at sacrifice. “V” stands for vehicle. Data are means ± SD from four mice per group. * *p* < 0.05, ** *p* < 0.01.

To evaluate the efficacy of WA, we assayed the tumor growth by subcutaneous implantation of cells, and found that the above results in vitro were confirmed in xenograft tumor models. Administration was performed according to the panel in Figure 2A. Because of the side effects of cisplatin, the weight of mice decreased in cisplatin-treatment groups at the late stage of therapy (Figure 2B). Compared with control group, silencing of ECM1 did not postpone tumor formation while retarded the growth of the tumor and increased the sensitivity to drugs (Figure 2C). Treatment with either WA or CDDP alone suppressed the growth of the ovarian carcinoma xenograft, but no significant differences were found between WA-treated tumors and cisplatin-treated tumors (Figure 2C–E). Treatment with both CDDP and WA further slowed the tumor growth, particularly at the late stage of tumor growth, and tumor volume was eventually reduced compared with the data from the first injection in ECM1 silencing A2780cis cells (Figure 2C). No significant differences of tumor weight were found between the CDDP/WA-treatment group and the vehicle treatment group in A2780cis xenograft-bearing mice. However, the combined treatment of A2780cis- or A2780cisi-induced xenograft mice with CDDP and WA significantly reduced tumor weights. The inhibitory rates of A2780cis- or A2780cisi-induced xenograft tumors treated with CDDP and WA were 92.87% or 98.03% compared with those of relative control cell-induced tumors treated with vehicles. Silencing of ECM1 also reversed drug resistance, and the inhibitory rates at the 20th day of CDDP and WA treatment groups in ECM1-silenced A2780cis xenograft-bearing mice were 68.71% and 75.84%, respectively (Figure 2D,E).These results indicate that WA reverses the cisplatin resistance, presumably through the altered expression of ECM1.

### 3.3. WA Inhibits the Cisplatin-Induced Activation of NF-κB/ECM1

**Figure 3:**
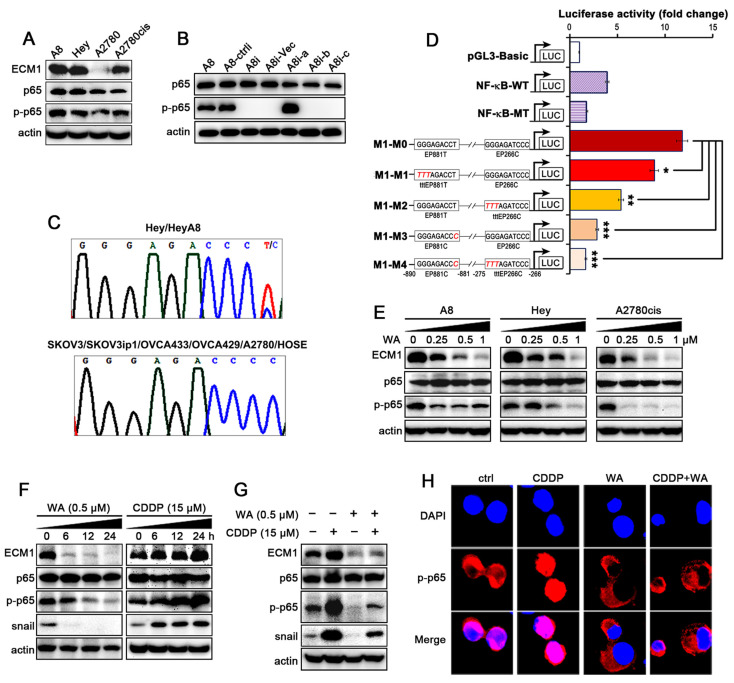
WA reverses ECM1 expression through the regulation of NF-κB activation. (**A**) Expression of ECM1, p-p65 and p65 in four ovarian cancer cell lines. (**B**) Detection of p65 phosphorylation in ECM1 silencing and overexpressing cells. (**C**) Sanger sequencing of ECM1 promoter; heterozygous T/C was present in Hey/A8 cells (upper panel), while homozygous C/C was present in the other cell lines (lower panel). (**D**) Luciferase activities of different promoters in A8 cells. ECM1 WT or MT promoters with two potential NF-κB p65 binding sites (EP881T/C and EP266C) are specifically indicated. HIV WT and mutant promoters known to bind to NF-κB are used as positive and negative controls, respectively. (**E**) Western blotting was used to detect ECM1, p-p65 and p65 in cells treated with different concentrations of WA. (**F**,**G**) A2780cis cells were treated with WA/cisplatin alone (**F**) or both WA and CDDP (**G**) for indicated times. The expression levels of ECM1, p-p65, p65 and snail were detected. Actin is shown as a protein-loading control. (**H**) The localization of p-p65 was detected by immunofluorescence. Means ± SD, *n* = 3. * *p* < 0.05, ** *p* < 0.01, *** *p* < 0.001.

A study reported that the function of ECM1 may be associated with NF-κB [35]. Therefore, we first tested ECM1, p65, phosphorylated p65 (p-p65) in different cell lines, and found that the high level of ECM1 was correlated with high expression of p-p65 in Hey-A8 (hereafter labeled as “A8”), Hey and A2780cis cells (Figure 3A and Figure S2A), and p-p65 was increased in cells overexpressing ECM1a, but not in cells expressing ECM1b, ECM1c in A8 cells (Figure 3B and Figure S2B).

To further determine how ECM1 is regulated in ovarian cancer cells, we analyzed the promoter region of ECM1 and found that two motifs located at nucleotides (nt) −890 to −881 (GGGagacCCC, named EP881C) and at −275 to −266 (GGGagatCCC, named EP266C) upstream of the transcription start site were potential p65 binding sites [32]. We also found an allelic difference among the ovarian cancer cell lines: a T in the −890 to −881 binding site (GGGagacCCT, hereafter named EP881T) in Hey and A8 (Figure 3C, upper panel), but a C in that position in the other five ovarian cancer cell lines and in HOSE cells (EP881C; Figure 3C, lower panel). The luciferase reporter assay proved that cells transfected with the ECM1 plasmid containing EP881T-EP266C (M1-M0) presented higher luciferase activity than those transfected with the wild-type (WT) or EP881C-EP266C luciferase vectors (M1-M3), whereas mutation (TTT substitution) in either EP881T (M1-M1) or EP266C (M1-M2) yielded intermediate activity (Figure 3D). TTT substitution in cells transfected with the EP881C-EP266C vector (M1-M4) resulted in the lowest luciferase activity (Figure 3D). These data suggest that the transcription of the ECM1 gene is initially regulated largely by NF-κB through the identified binding sites; EP881C-EP266C have a moderate affinity for NF-κB binding, whereas EP881T-EP266C have a higher affinity for NF-κB than EP881C-EP266C in triggering ECM1 gene transcription. Therefore, it is confirmed that NF-κB is the transcription factor of ECM1, and the ECM1 gene could be regulated by NF-κB through EP881C/T-EP266C binding sites.

To determine whether WA regulates NF-κB/ECM1 signaling, we incubated A8, Hey, and A2780cis cell lines with 0.25, 0.5 and 1 µM WA, and found that WA inhibited the expression of ECM1 and p-p65 in a dose-dependent manner (Figure 3E and Figure S2C). When we further treated A2780cis cells with 0.5 µM WA at 0, 6, 12, 24 h, respectively, the expression of ECM1, p-p65 and snail was decreased in a time-dependent manner, whereas the expression of these proteins was increased in cells treated with 15 µM cisplatin (Figure 3F and Figure S2D). The combined treatment with WA inhibited the expression of these proteins elevated by cisplatin treatment (Figure 3G and Figure S2E). As the phosphorylation of p65 at Ser536 is associated with its transcriptional activity, we performed immunofluorescence (IF) assays and found that WA attenuated the phosphorylation and nuclear localization of NF-κB p65, whereas treatment with cisplatin alone induced the p-p65 nuclear translocation (Figure 3H). These results indicate that WA inhibits the phosphorylation of NF-κB to block the expression of ECM1 and reverses the cisplatin-induced activation of NF-κB/ECM1.

### 3.4. The Secreted ECM1 Induces Malignant Transformation of NFs

Tumor microenvironment consists of the tumor bulk and supporting cells. Cancer-associated fibroblasts (CAFs), transformed from normal fibroblasts (NFs), are one of the most significant components in the tumor microenvironment [9]. CAFs-mediated extracellular matrix (ECM) remodeling may affect the delivery of chemotherapeutic drugs so as to confer cancer tissue chemo-resistance [7].

**Figure 4:**
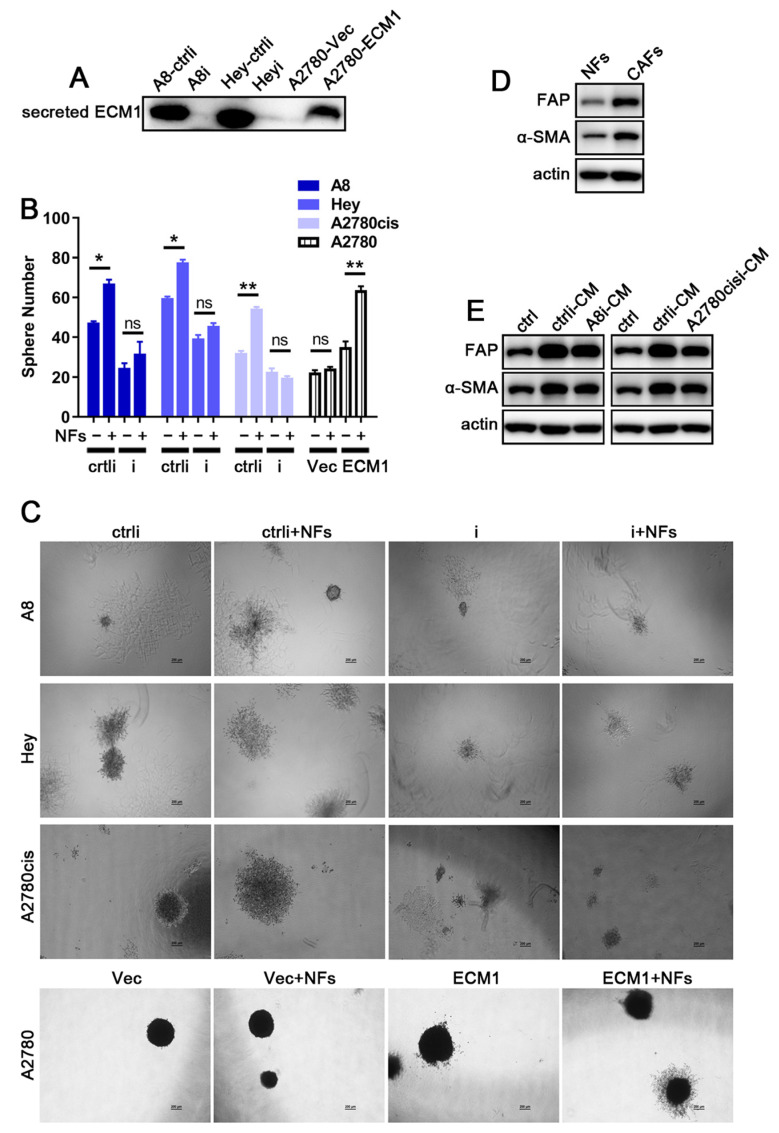
Secreted ECM1 induces NFs appearing with malignant phenotypes. (**A**) Secreted ECM1 in conditioned medium (CM) of ovarian cancer cells. (**B**,**C**) Ovarian cancer cells were co-cultured with or without NFs in 3D spheroid formation assay. The spheres were observed under a microscope. Scale bars: 200 µm. (**D**) The expression levels of FAP and α-SMA in CAFs and NFs. (**E**) NFs were incubated with CM of A8 or A2780cis cell lines for 48 h, and FAP and α-SMA of NFs were detected. Actin is shown as protein-loading control. Means ± SD, *n* = 3. ns, not significant, * *p* < 0.05, ** *p* < 0.01.

Because ECM1 is a secreted protein, to assess the effect of ECM1 on the tumor microenvironment, we first detected the secreted ECM1 in CMs of different cancer cells, and found that ECM1 was highly secreted in A8, Hey, and A2780-ECM1 cells compared with in ECM1 knockdown and A2780 cells (Figure 4A and Figure S3A). In three-dimensional (3D) culture models, co-culture of high ECM1-expression cancer cells (A8-ctrli, Hey-ctrli, A2780cis-ctrli, A2780-ECM1) with NFs formed more and larger spheroids than without NFs. However, co-culture of low ECM1-expression cancer cells (A8i, Heyi, A2780cisi, A2780-Vec) with NFs failed to induce more and larger spheroids compared than without NFs (Figure 4B and Figure S3C). These results indicate that ovarian cancer cells and NFs may interact through the secreted ECM1 protein. We further found that the expression level of fibroblast activation protein (FAP) and α-smooth muscle actin (α-SMA) was higher in CAFs than in NFs (Figure 4D and Figure S3B). We then incubated NFs with the CMs of A8i and A2780cisi cells and relevant control cells. We found that FAP and α-SMA were increased in NFs treated with the CMs from A8-ctrli and A2780cis-ctrli cells compared with in those treated with the CM from normal human ovarian epithelial HOSE cells (control, hereafter labeled ‘ctrl’). However, the CMs from ECM1-silenced A8i and A2780cisi cells decreased the expression of FAP and α-SMA compared with those from high ECM1-expression cells (Figure 4E and Figure S3C). These results suggest that the secreted ECM1 from cancer cells activates NFs, which enables NFs to appear with characteristics of CAFs in both phenotypes and biological behaviors.

### 3.5. WA Inhibits IKKs/IκBα Phosphorylation

**Figure 5:**
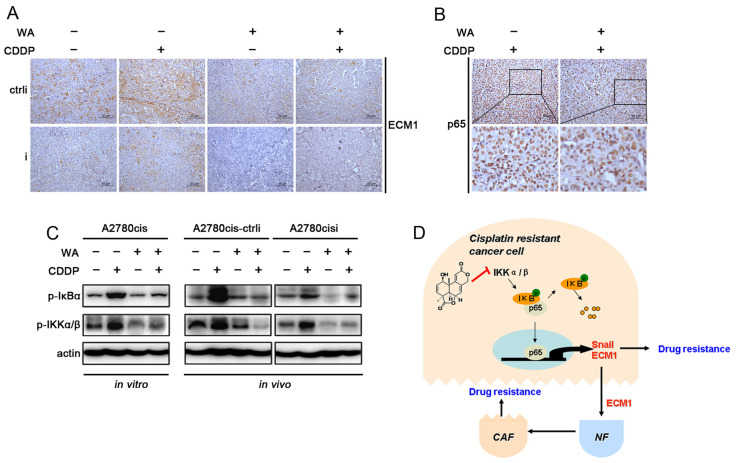
WA suppresses the activation of NF-κB signal pathway. Representative photomicrographs of IHC analysis for ECM1 (**A**) and p65 (**B**). Scale bars: 50 µm. (**C**) Changes of phospho-IκBα (p-IκBα) and phospho-IKKα/β (p-IKKα/β) in A2780cis cells administrated with WA, CDDP or combination in vitro and in vivo. Actin is shown as protein-loading control. (**D**) A schematic diagram shows that wentilactone A inhibits the activation of IKK/IκB to reverse cisplatin resistance induced by the NF-κB/ECM1/Microenvironment axis.

To validate the previous results in vitro, we analyzed the expression of ECM1 and p65 in xenograft tumor tissues derived from animal experiments, and found that WA treatment largely suppressed the expression of ECM1 induced by cisplatin (Figure 5A). Especially in A2780cisi cells, there was no complete cell structure after combined treatment with WA, therefore no ECM1 was detected in vivo. Moreover, the combined treatment with WA decreased the expression of p65, and partially induced p65 nuclear localization compared with treatment using cisplatin alone (Figure 5B). To investigate the upstream molecules of the WA-inhibited NF-κB signaling, we further detected the phosphorylation of IKKα/β and IκBα by western blot, and found that the changes of phospho-IκBα (p-IκBα) and phospho-IKKα/β (p-IKKα/β) were consistent with those of p-p65 and ECM1 in A2780cis cell line (Figure 5C and Figure S4A, in vitro). In mice tumor tissues, WA also inhibited the phosphorylation of IκBα and IKKα/β (Figure 5C and Figure S4B, in vivo), indicating that WA regulates NF-κB through phosphorylation of the upstream IKK/IκB molecules.

## 4. Discussion

The extracellular matrix (ECM) participating in tumorigenesis, cancer metastasis, chemo-resistance, and tumor microenvironment remodeling [36,37]. It contains molecules that may be suitable targets for cancer treatment. ECM protein-1 (ECM1) is of great interest among ECM components. We previously reported that the heterogeneous nuclear ribonucleoprotein-like (hnRNPLL) protein, the Serine/Arginine Splicing Factor (SRSF) 1 and 6 actively participate in the alternative mRNA splicing of ECM1 isoforms, however, we did not investigate the transcriptional regulation of ECM1 [27]. In mammalian cells, the NF-κB p50/p65 (RelA) heterodimer may participate in the transcription of most genes [38]. Most of NF-κB-regulated genes commonly contain at least a consensus nucleotide sequence in the promoter regions to which NF-κB can bind. For example, GGGRNNYCCC (where R denotes a purine, N denotes any nucleotide, and Y denotes a pyrimidine) is the specific consensus sequence to which p65 binds to [32]. It has not been reported yet whether the transcription of ECM1 can be mainly regulated by NF-κB p65 through binding to the consensus sequence in ECM1 promoter region. In this study, we first identified that two binding sequences of NF-κB p65 in the ECM1 promoter were responsible for ECM1 transcription by mutagenesis and luciferase assays. Interestingly, NF-κB may function as both a trigger and a linker to mediate the ECM1a-associated tumorigenic signaling, because ECM1a also promotes NF-κB p65 phosphorylation (Figure 3B).

TCAGCCTCCCTCACATGGGAGACCC[C/T]AACCCAGCTGACAATGTGGAGCCCC, which contains EP881C/T located at chromosome 1:150507129, was named rs114293104 in the ECM1 and TARS2 genes. Genomic sequencing data indicates that SNP might have a race-based difference (Table 2). Interestingly, in the presence of the alternative allelic change from GGGagacCCC (EP881C) to GGGagacCCT (EP881T), the luciferase activity of EP881T was five-fold higher than the activity of EP881C. Although we did not find EP881T in more than six additional ovarian cancer cell lines, the genome sequencing data from the GWS database indicate that this SNP is a race-based alteration. Moreover, no association of this SNP with ovarian cancer or other types of cancer from The Cancer Genome Atlas (TCGA) database has been reported to date. Therefore, it requires further investigation whether this SNP indicates any cancer risk (including ovarian cancer) at the genetic level and whether the mutation of −881T in specific populations can be used as a diagnostic marker during cancer development.

Nuclear NF-κB activates anti-apoptotic genes, cell growth factors, multi-drug resistance genes, angiogenesis-related genes, and genes related to cell adhesion and metastasis, etc. at the transcriptional level, which leads to uncontrolled proliferation, apoptosis escape, and chemo-resistance of malignant cancer cells [39,40,41,42,43]. Therefore, as a potential target for treatment of malignant tumors, NF-κB is related to the prognosis of cancer [44]. However, many first-line conventional chemotherapeutic drugs, including cisplatin, always induce the activation of bypass effectors, e.g., NF-κB and snail, which weakens drug efficacy and eventually leads to treatment failure and tumor recurrence [4,6,45]. Currently, the inhibitors of NF-κB mainly include IKKs activity inhibitors, IκB phosphorylation inhibitors, and NF-κB transcription complex inhibitors, etc. Among them, no data is reported on clinical efficacy because of toxicity and side effects [5,43,44]. However, candidate drugs such as CDDO-Me, selicicalib and curcuminin clinical trials were proven to be correlated with NF-κB signal [46]. Curcumin can reverse cisplatin resistance and cooperate with cisplatin in a variety of tumor models, especially ovarian cancer [47,48]. Therefore, it can be concluded that NF-κB inhibitors from natural products may be promising clinical adjuvants to overcome cisplatin resistance. We prove that WA as a natural NF-κB inhibitor is effective in vitro and in vivo with less toxic effects. In this study, WA reverses cisplatin resistance through inhibiting the phosphorylation of NF-κB to block the expression of ECM1 induced by cisplatin. Morevoer, our data shows that WA reverses the elevation of the EMT transcription factor snail which may also directly contribute to cisplatin resistance in ovarian cancer (Figure 3F,G) [49].

It is known that the activation of the IKK (IκB-kinase) complex (IKKα-IKKβ-IKKγ) can induce the phosphorylation and ubiquitination of IκBα. NF-κB is then phosphorylated and translocated into nucleus to activate the NF-κB responsive genes, which is called the classical pathway of NF-κB activation [50]. WA inhibits the activation of NF-κB through a classical pathway induced by cisplatin, which further blocks the NF-κB downstream effectors such as ECM1. ECM1 as a chemo-resistance effector is reported to promote trastuzumab resistance and the PKM2-mediated Warburg effect through the activation of epidermal growth factor [17,51]. Our previous study indicates that the higher expression of ECM1b is associated with good survival, while higher expression of ECM1a is associated with poor survival [27]. Therefore, in this study we show that high levels of ECM1a is associated with cisplatin resistance; low ECM1a confers the higher sensitivity to cisplatin resistant cells.

Moreover, secretory ECM1 from cancer cells can activate NFs to display characteristics of CAFs, which promotes tumor progression and cisplatin resistance. CAFs are the most common type of stromal cells in tumor microenvironment. The abnormal activation of CAFs is closely related to the malignant transformation of cancer cells [52,53]. It is reported that ECM remodeling mediated by CAFs may affect the delivery of chemotherapy drugs, thereby resulting in chemotherapeutic failure [54,55]. NFs are the main source of CAFs. It is critical to understand how NFs are activated and transformed to CAFs, thereby supporting cancer cells’ growth and micro-metastasis. However, this activation process is not yet understood. As a secretory protein, ECM1 may function through transmitting cell signals [56]. Current studies only show that high levels of ECM1 are detected in the plasma of tumor patients [57,58], but the function of ECM1 in tumor interstitial microenvironment is not yet reported. Our study shows that ECM1 activates NFs to transform cells with characteristics of CAFs, which induces tumor microenvironment reconstruction and transmits drug resistance signals between cancer cells and tumor microenvironmental cells.

All of our data indicate that NF-κB p65 regulates ECM1 expression and activates ECM1 at the transcriptional level. A high level of ECM1 in stroma can induce the malignant transformation of NFs and transfer tumor signals to promote tumor progression. WA reduces the expression of ECM1 through the IKK/IκB/NF-κB signaling pathway to reverse the cisplatin resistance of cancer cells and to facilitate the drug resistance of the tumor microenvironment, which provides a rationale for the development of WA as a natural chemotherapeutic agent against cisplatin resistant ovarian cancer (Figure 5D).

## 5. Conclusions

ECM1, as an effector regulated by NF-κB transcription, is associated with cisplatin resistance. High levels of ECM1 in stroma can induce the malignant transformation of NFs and transfer tumor signals to promote tumor progression. Therefore, ECM1 could be a selective target for cancer therapy. Wentilactone A reverses the NFκB/ECM1 signaling-induced cisplatin resistance through the inhibition of IKK/IκB in ovarian cancer cells, which provides the possibility of cooperation with cisplatin to improve the cancer patient survival.

## Figures and Tables

**Table 1:** Primers used in ECM1 promoter cloning.

N	Names	Sequences and Notes	Purposes
1	ECM1-P-FW	5′-GTGGAGCTACAGAACACGAGGGTC-3′	ECM1 promoter cloning
2	ECM1-P-RV	5′-CACATCCAAACAGCTACAGCTTCCC-3′	ECM1 promoter cloning
3	tttEP881T-FW	5′-cctccctcacatTTTagaccctaacccagctgac-3′	ECM1 promoter mutation
4	tttEP881T-RV	5′-gtcagctgggttagggtctAAAatgtgagggagg-3′	ECM1 promoter mutation
5	tttEP266C-FW	5′-cacactggtagTTTagatcccttggataggtt-3′	ECM1 promoter mutation
6	tttEP266C-RV	5′-aacctatccaagggatctAAActaccagtgtg-3′	ECM1 promoter mutation
7	EP881C-FW	5′-cctccctcacatgggagacccCaacccagctgac-3′	ECM1 promoter mutation
8	EP881C-RV	5′-gtcagctgggttGgggtctcccatgtgagggagg-3′	ECM1 promoter mutation

**Table 2:** Alteration of rs114293104 in different populations.

Population	Chrom.SampleCnt.	Source	C	T
EAS *	1008	AF	1	0
EUR	1006	AF	0.9811	0.0189
AFR	1322	AF	0.9985	0.0015
AMR	694	AF	0.9914	0.0086
SAS	978	AF	1	0

* EAS, EAS, East Asian; EUR, European; AFR, African; AMR, Ad Mixed American; SAS, South Asian

## Data Availability

The rest datasets used or analyzed during the current study are available from the corresponding author on reasonable request.

## Supplementary Materials

The following supporting information can be downloaded at: https://www.mdpi.com/article/10.3390/nu14183790/s1, Figure S1: Quantification of ECM1 protein normalized to actin in Figure 1E; Figure S2: Quantification of protein levels in Figure 3; Figure S3: Quantification of protein levels in Figure 4; Figure S4: Quantification of protein levels in Figure 5.
