# Supplementary material for: Wentilactone A Reverses the NF-κB/ECM1 Signaling-Induced Cisplatin Resistance through Inhibition of IKK/IκB in Ovarian Cancer Cells
Source: Nutrients. 2022 Sep 14;14(18):3790. doi: 10.3390/nu14183790 (PMC9504226; doi:10.3390/nu14183790)
Supplement: Supplementary file 1 [file nutrients-14-03790-s001.zip › nutrients-1830118-supplementary.pdf]

Supplementary Data

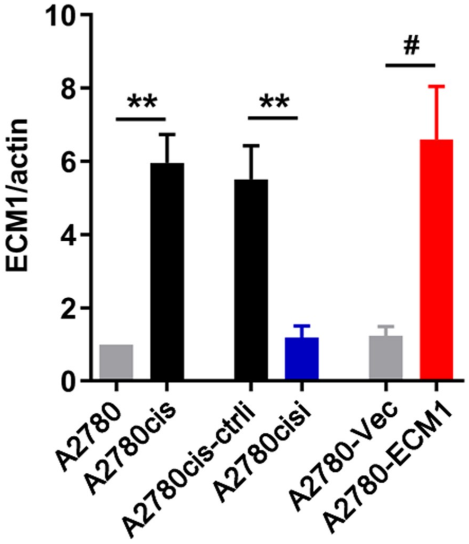

Figure S1. Quantification of ECM1 protein normalized to actin in Figure 1E. ECM1 expression in A2780, A2780cis cell lines and their derivatives. The data are shown as means ± SD, n = 3 independent repeats. ** $p < 0.01$, # $p < 0.001$ *vs*. control group.

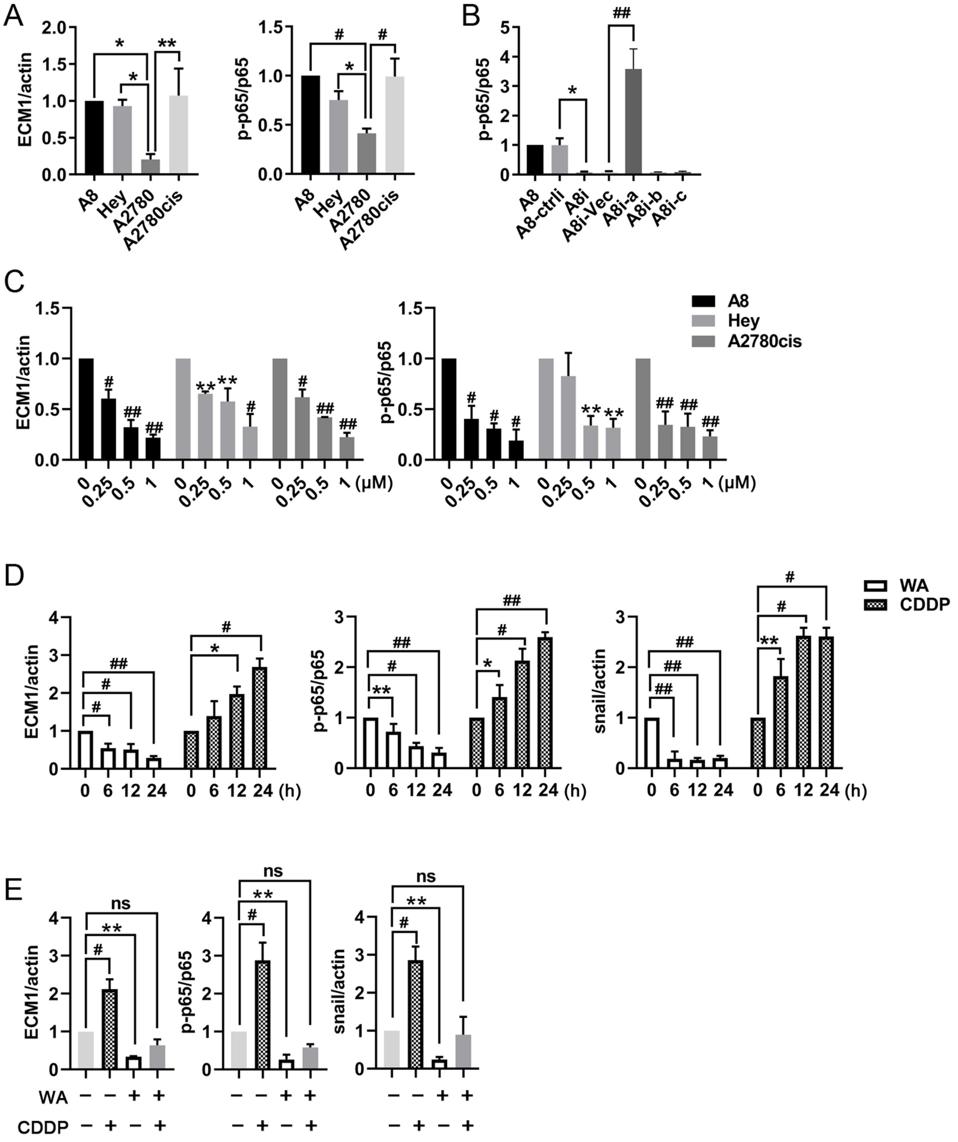

Figure S2. Quantification of protein levels in Figure 3. (A) The levels of ECM1 and p-p65 in four ovarian cancer cell lines. (B) Detection of p65 phosphorylation in ECM1 silencing and overexpressing cells. (C) The levels of ECM1 and p-p65 after treatment with different concentration of WA in A8, Hey and A2780cis cells. (D and E) The levels of ECM1, p-p65 and snail after the treatment of A2780cis cells with WA/cisplatin alone (D) or both WA and CDDP (E). The data are shown as means ± SD, n = 3 independent repeats. *ns*, not significant, * $p < 0.05$, ** $p < 0.01$, # $p < 0.001$, ## $p < 0.0001$.

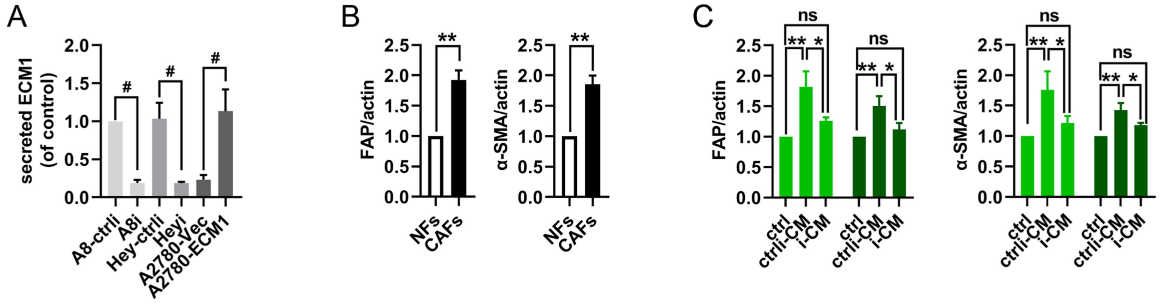

Figure S3. Quantification of protein levels in Figure 4. (A) The levels of secreted ECM1 in conditioned medium (CM) of different ovarian cancer cells. (B) The expression levels of FAP and α-SMA in CAFs and NFs. (C) The expression of FAP and α-SMA in NFs incubated with CM of HOSE (control), A8 or A2780cis cell lines for 48 h. The data are shown as means ± SD, $n = 3$ independent repeats. *ns*, not significant, * $p < 0.05$, ** $p < 0.01$, # $p < 0.001$.

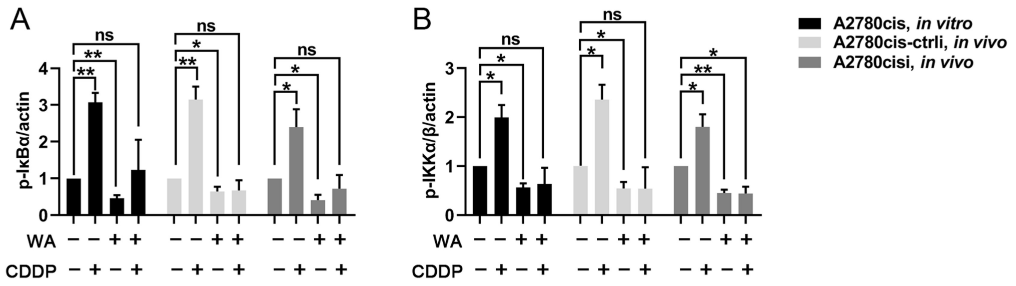

Figure S4. Quantification of protein levels in Figure 5. (A and B) Western blotting analysis of p-IκBα (A) and p-IKKα/β (B) *in vitro* and *in vivo*. The data are shown as means ± SD, $n = 3$ independent repeats. *ns*, not significant, * $p < 0.05$, ** $p < 0.01$.
